# Characterizing Surface Deformation of the Earthquake-Induced Daguangbao Landslide by Combining Satellite- and Ground-Based InSAR

**DOI:** 10.3390/s25010066

**Published:** 2024-12-26

**Authors:** Xiaomeng Wang, Wenjun Zhang, Jialun Cai, Xiaowen Wang, Zhouhang Wu, Jing Fan, Yitong Yao, Binlin Deng

**Affiliations:** 1 School of Environment and Resources, Southwest University of Science and Technology, Mianyang 621010, China; wangxiaomeng@mails.swust.edu.cn (X.W.); shouyeren@mails.swust.edu.cn (Z.W.); fanjing@mails.swust.edu.cn (J.F.); yaoyitong@mails.swust.edu.cn (Y.Y.); dengxiaolin@mails.swust.edu.cn (B.D.); 2 Mianyang Science and Technology City Division, The National Remote Sensing Center of China, Mianyang 621010, China; 3 Faculty of Geosciences and Engineering, Southwest Jiaotong University, Chengdu 610031, China; insarwxw@gmail.com

**Keywords:** interferometric synthetic aperture radar (InSAR), ground-based radar (GB-SAR), Daguangbao landslide, downslope displacement transformation

## Abstract

The Daguangbao landslide (DGBL), triggered by the 2008 Wenchuan earthquake, is a rare instance of super-giant landslides globally. The post-earthquake evolution of the DGBL has garnered significant attention in recent years; however, its deformation patterns remain poorly characterized owing to the complex local topography. In this study, we present the first observations of the surface dynamics of DGBL by integrating satellite- and ground-based InSAR data complemented by kinematic interpretation using a LiDAR-derived Digital Surface Model (DSM). The results indicate that the maximum line-of-sight (LOS) displacement velocity obtained from satellite InSAR is approximately 80.9 mm/year between 1 January 2021, and 30 December 2023, with downslope displacement velocities ranging from −60.5 mm/year to 69.5 mm/year. Ground-based SAR (GB-SAR) enhances satellite observations by detecting localized apparent deformation at the rear edge of the landslide, with LOS displacement velocities reaching up to 1.5 mm/h. Our analysis suggests that steep and rugged terrain, combined with fragile and densely jointed lithology, are the primary factors contributing to the ongoing deformation of the landslide. The findings of this study demonstrate the effectiveness of combining satellite- and ground-based InSAR systems, highlighting their complementary role in interpreting complex landslide deformations.

## 1. Introduction

Earthquake-induced landslides are among the most severe geological hazards, capable of causing significant casualties and extensive property damage—sometimes even surpassing the direct impact of the earthquake itself [1,2]. Studying the spatial and temporal evolution characteristics of seismic landslides can enhance early warning systems and improve our understanding of their motion mechanisms. On 12 May 2008, a powerful earthquake (Mw 8.1) with its epicenter in Wenchuan County, Sichuan Province, struck the Longmenshan fault zone at the eastern edge of the Qinghai–Tibet Plateau in China. This earthquake triggered numerous secondary geological hazards, including mudslides and landslides [3]. Among these, the DGBL, with a cumulative material volume approaching 1 × 10^9^ m^3^, stands as the largest landslide triggered by the Wenchuan earthquake. It serves as a unique example of a mega-scale landslide on a global scale. Monitoring the surface deformation of the DGBL is crucial for understanding its evolution and assessing its stability in the aftermath of the earthquake.

Following the DGBL, numerous researchers have employed various techniques to characterize its geometric features, thickness, accumulation volume, and surface movement. Typical studies include (1) analyzing the causes of the discontinuous geological features of the landslide using field photographs, remote sensing images, and rock mass structural surface attitude data [4]; (2) creating a three-dimensional solid model of the ground surface with UAV aerial imagery and subsequently calculating the maximum thickness and volume of the landslide accumulation [5]; and (3) utilizing satellite interferometric SAR (InSAR) to monitor the deformation characteristics of the landslide both before and after its occurrence. Among these, satellite InSAR monitoring data indicate that the DGBL has remained active since the earthquake. Chen et al. mapped the surface deformation of DGBL using the Permanent Scatterer InSAR with the ALOS/PALSAR images acquired between 2008 and 2011. They estimated a displacement rate ranging from −300 to 300 mm/year following the earthquake [6]. Dai et al. analyzed Sentinel-1 images acquired from 2015 to 2016 and obtained an annual displacement rate ranging between −80 and 40 mm/year for the DGBL in the SAR LOS direction [7]. Yu et al. processed 65 Sentinel-1A images of the DGBL from 2016 to 2018 using the IPTA method, revealing a maximum LOS deformation rate of about −50 mm/year [8]. Additionally, Luo et al. utilized both ascending and descending Sentinel-1A images from November 2015 to June 2020 to assess vertical and east–west two-dimensional deformation, identifying a maximum vertical displacement rate of about 80 mm/year and east–west displacement of about 35 mm/year [9].

It can be seen that previous studies utilizing satellite InSAR have yielded inconsistent deformation results concerning the kinematics of the DGBL. These discrepancies may arise from the continuous movement of the landslide at varying rates over time or from differences in the data processing techniques employed by researchers. Additionally, satellite-based methods are limited by the single looking geometry, the complexity of the terrain, and the dynamic nature of the landslide’s movement.

To overcome these limitations and improve monitoring accuracy, integrating satellite observations with ground-based methods is crucial. Ground-based SAR (GB-SAR) is a robust technology that provides high-resolution deformation measurements with sub-millimeter accuracy and is widely applied in engineering and geological fields [10,11]. The GB-SAR system operates on the same principles as space-based sensors to monitor ground deformation phenomena. However, the spatial coverage of satellite data is constrained by the imaging geometry of SAR sensors, such as layover and shadow effects. In contrast, GB-SAR can be positioned in front of steep slopes, which are often not visible from space-borne platforms [12]. Consequently, the integration of these technologies enables the acquisition of high-precision measurements with enhanced spatial and temporal resolution for ground displacement information.

This study aims to elucidate the detailed deformation characteristics of the DGBL by integrating both ground-based and satellite-based InSAR measurements. Initially, a time series of deformation in the LOS direction of the DGBL is obtained using the small baseline subset (SBAS) technique with Sentinel-1 data from both ascending and descending orbits, covering the period from 1 January 2021, to 30 December 2023. Subsequently, the downslope displacement features of the DGBL are mapped by projecting the displacements in the SAR LOS direction to the downslope direction. The deformation rate of the extensive landslide is then mapped along its slope direction. Furthermore, a comprehensive analysis of the temporal and spatial deformation characteristics of the DGBL is conducted by combining deformation results obtained with satellite- and ground-based InSAR. The findings of this study contribute to a deeper understanding of the mechanisms underlying landslide evolution, particularly for those induced by seismic activities.

## 2. Study Area and Datasets

### 2.1. Study Area

**Figure 1:**
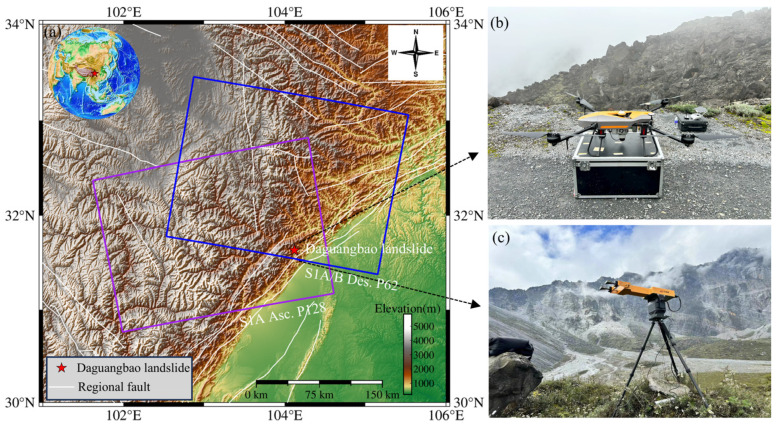
(**a**) The geographical location of the DGBL and the coverage of the SAR image. The rectangular window indicates the coverage of Sentinel-1 data, and the red pentagram marks the location of the DGBL. (**b**) The UAV equipped with the AU20 multi-platform laser scanning system is primarily used to collect external auxiliary data such as DSM and DOM. (**c**) Ground-based radar field monitoring site (from 15 October to 17 October 2024).

DGBL is geographically located at longitude 104°07′0.2″ E, latitude 31°38′19.8″ N, in Anzhou District, Mianyang City, Sichuan Province, China, and is situated within the Longmenshan fault zone in the mountainous area of northwestern Chengdu, which is part of the eastern edge of the Tibetan Plateau [13]. Following the 2008 Wenchuan earthquake, the elevation of the region decreased from 3047 to 2964 m. The rock detritus traversed the Huangdongzi Valley at the base of the slope, advancing toward Pingliangzi on the opposite side, and extending longitudinally (in the northeast-east-east [NEE] direction) for 4.2 km. The maximum lateral width along Huangdongzi Valley reached 3.2 km, with the primary accumulation area spanning 2.2 km. The landslide dam achieved a height of 690 m, with an estimated volume of approximately 1.2 × 10^9^ m^3^ [14,15,16]. The study area predominantly comprises Paleozoic to Cenozoic sedimentary and metamorphic rocks, characterized by fractured rock layers and well-developed faults. This brittle geological foundation is conducive to landslide occurrences [17,18]. Figure 1 provides an overview of the study area.

**Figure 2:**
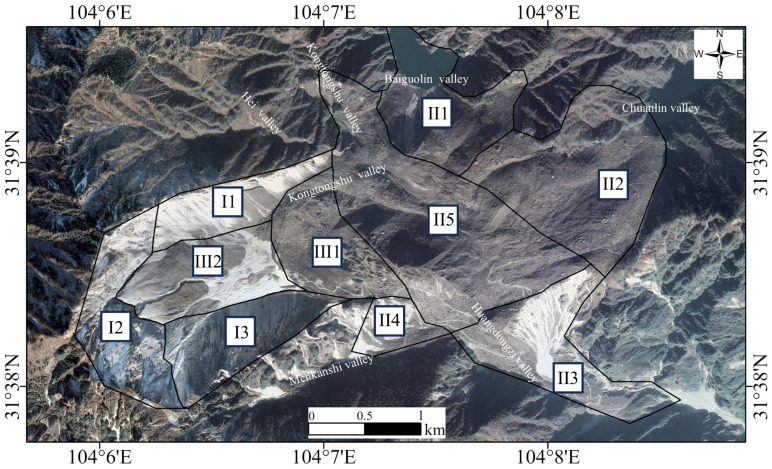
Zoning map of DGBL based on satellite imagery. I1 represents the Northern Scarp Zone; I2 is the Rear Scarp Zone; and I3 is the Southern Scarp Zone. II1 and II2 correspond to the Baiguolin and Chuanlin Gully Barrier Lake Zones, respectively. II3 and II4 are the Huangdongzi and Menkanshi Gully Accumulation Zones. II5 represents the Main Accumulation Zone. III1 refers to the Secondary Slide Mass, while III2 is the Collapse Accumulation Zone.

Figure 2 presents a zoning map of the DGBL. The landslide area is categorized into three primary zones: Landslide Scarp Zone (I), Main Landslide Accumulation Zone (II), and Secondary Accumulation Zone (III). Zone I is further divided into three sub-areas: I1, the Northern Scarp Zone, consisting of dolomite and slate with significant rockfall deposits; I2, the Rear Scarp Zone, marked by distinct tensile cracks; and I3, the Southern Scarp Zone, situated along the southern sliding surface and composed of rocks from the Sinian Dengying Formation. Zone II comprises five sub-areas: II1 and II2, which are barrier lakes created by the rock detritus in Baiguolin and Chuanlin Gullies; II3 and II4, which are accumulation zones in Huangdongzi and Menkanshi Gullies; and II5, the Main Accumulation Zone, serving as the primary deposition site for landslide materials. Zone III contains two sub-areas: III1, the Secondary Slide Mass, which detached from the main landslide body and settled in this zone, and III2, the Collapse Accumulation Zone, consisting of loose and fragmented rock debris from the rear scarp [15].

### 2.2. SAR Datasets

In this study, the DGBL is located in a mountainous area with large terrain fluctuations, and its imaging in SAR images is often affected by terrain slope. In the observed geometry of the ascending and descending orbits, the landslide area exhibited different degrees of geometric distortion. By comprehensively analyzing the data of the two orbits, the ascending and descending orbits can effectively complement each other, reduce the influence of geometric distortion on the monitoring results, and improve the accuracy and reliability of monitoring. We collected 80 ascending Sentinel-1A images and 111 descending Sentinel-1A/B images acquired between 1 January 2021, and 30 December 2023. Because of the slope and orientation of the DGBL, the ascending images are less affected by SAR imaging distortions than the descending images. Therefore, to achieve more accurate data results, we utilized both Sentinel-1A and Sentinel-1B satellites for the descending orbit [19]. The coverage of the SAR images is illustrated in Figure 1, and the basic parameters of the images are listed in Table 1. Additionally, we used the Copernicus Digital Elevation Model (DEM), with a 30 m resolution, to eliminate the interferometric topographic phase [20,21,22].

### 2.3. GB-SAR Datasets

To inspect the detailed surface deformation pattern of DGBL, we implemented a field campaign using the portable micro-deformation monitoring radar MPDMR-HSB, built by Fangxiangtu Technology Co., Ltd., Inner Mongolia, China. The GB-SAR system is an arc-scanning radar that collects time series echo data from the observation area through multiple reciprocal-rotating scans. This method achieves sub-millimeter accuracy in deformation monitoring by utilizing interferometric techniques [23,24]. The arc-type GB-SAR system can achieve 360° full coverage monitoring at distances ranging from 100 to 5000 m, with a collection interval of 6 min, providing a higher azimuth resolution than traditional rail-type GB-SAR.

The GB-SAR system was installed in a stable area with unobstructed open field vision in front of the DGBL (Figure 1c). The scanning area fully covered the rear edge of the landslide. During the observation period from 16:00 on 15 October to 12:00 on 17 October 2023, the antenna was set to a horizontal angle of 60°. A total of 383 SAR images were obtained. The basic settings of the GB-SAR system are presented in Table 2. During the observation period, unfavorable weather conditions such as thick clouds or rainfall were not recorded.

### 2.4. Ancillary Datasets

**Figure 3:**
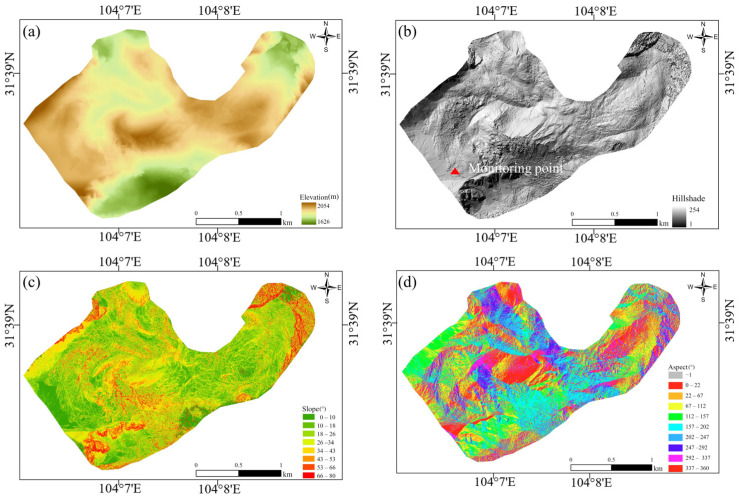
Aerial LiDAR remote sensing interpretation map of the DGBL. (**a**) High-resolution Digital Elevation Model (DEM). (**b**) Hillshade map, with red triangles indicating the locations of GB-SAR monitoring sites. (**c**) Slope map. (**d**) Aspect map.

Satellite- and ground-based InSAR deformation measurements are effective in revealing the characteristics of slow-moving landslides, providing valuable insights for stability assessments. In addition to the InSAR data, we also collected the LiDAR-derived Digital Surface Model (DSM) [25] of the DGBL. We employed the AU20 multi-platform laser scanning system to collect LiDAR data for the visual mapping of the detailed topographic features of the landslides. The processed data are then used to construct a Digital Surface Model (DSM) and a Digital Orthophoto Map (DOM), as well as to generate shaded relief maps, slope maps, and aspect maps. Figure 3 shows the aerial LiDAR remote sensing interpretation map of the DGBL area. By combining the topographic data and InSAR-derived surface displacements, we conducted a detailed analysis of the kinematic evolution and mechanisms of DGBL [26,27].

## 3. Methods

**Figure 4:**
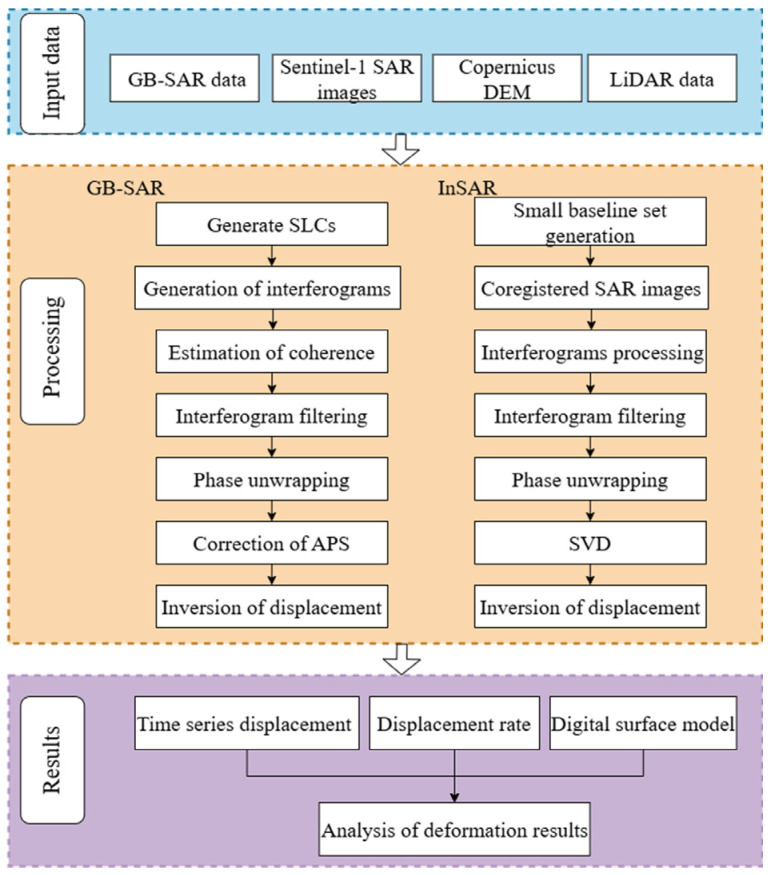
Data processing flow diagram.

Figure 4 illustrates the data processing workflow, which integrates the satellite- and GB-SAR InSAR observations utilized in this study.

### 3.1. Satellite SAR Data Processing

We employed the small baseline subsets InSAR (SBAS-InSAR) technique to process the SAR images. The SBAS method can provide more continuous deformation measurements across both temporal and spatial dimensions, is suitable for monitoring both slow linear and nonlinear deformation of long sequences, and is widely used in monitoring landslides [28,29]. The images acquired on 1 January 2021, and 30 December 2023, were designated as the reference images for the ascending and descending paths, respectively. Given that the DGBL is characterized by complex terrain and dense vegetation coverage, to ensure sufficient interferometric image pairs and limit the impact of the temporal and spatial decorrelation, a reasonably short perpendicular baseline and temporal baseline was set to comprise the interferometric image pairs with a small baseline subset. In addition, selecting interferometric image pairs with shorter baselines can effectively reduce the phase errors caused by excessively long baselines, thereby improving the accuracy of surface deformation monitoring. We set the temporal baseline threshold of 60 days and the spatial baseline threshold of 200 m for image pair selection [30]. We generated ascending and descending interferometric phase maps using the ISCE 2.6.0 software [31]. To reduce speckle noise, we applied a multi-look of 5 × 1 (range × azimuth) to the interferograms. Additionally, we used the Goldstein filter to suppress noise and employed the minimum cost flow method for phase unwrapping [32]. By removing the interferograms with mean coherence lower than 0.6, we finally obtained 184 ascending and 305 descending interferograms for further displacement time series analysis. The selected interferograms were then used to estimate the ground surface displacement time series and velocity using the MintPy 1.5.0 software [33]. We used the GACOS data to eliminate atmospheric delay errors to improve the accuracy of the deformation measurement [34]. Finally, we obtained the surface deformation velocities using the singular value decomposition (SVD) method [35].

### 3.2. GB-SAR Data Processing

The GB-SAR data processing is comparable to satellite InSAR time series analysis. However, unlike satellite interferometry, ground-based interferometry typically employs a zero-baseline approach; that is, all images are captured from the same location, which enhances the precision of deformation measurements [36]. First, we begin with image registration, where the corresponding pixels in the images match the same ground locations, thereby achieving registration accuracy at the subpixel level. We obtained a total of 382 phase interferograms by computing the phase differences from multiple acquisitions. We then employed the Goldstein filter for adaptive filtering and to reduce the noise in the interferograms. After converting the obtained phase values to the desired true phase values, an atmospheric model was established for the corresponding corrections to improve measurement accuracy. Finally, by analyzing the time series data, geocoding was performed to convert the GB-SAR data processing results from the SAR coordinate system to the geographic coordinate system, thereby obtaining the displacement velocity field in the SAR LOS direction [37,38].

### 3.3. Downslope Displacement Calculation

Debris on the landslide surface moved downward along the landslide face under the action of gravity. The displacement of the landslide mass mainly occurred on the landslide surface. The intensity and direction of a landslide are significantly influenced by the gradient and slope orientation of the landslide surface, which are crucial factors for the material flow of the landslide [39]. To more effectively study the displacement and deformation situation downslope, we converted the satellite InSAR’s LOS measurements to the downslope direction. Thus, we established a landslide migration coordinate system based on the migration patterns of the side aspect and slope direction (Figure 4) [40,41]. This system assumes that every target point on the landslide surface moves in a specific direction along a unit vector $\hat{u}$. Note that the downslope movement is defined as positive, reflecting the characteristic motion of the landslide parallel to the slope surface.

**Figure 5:**
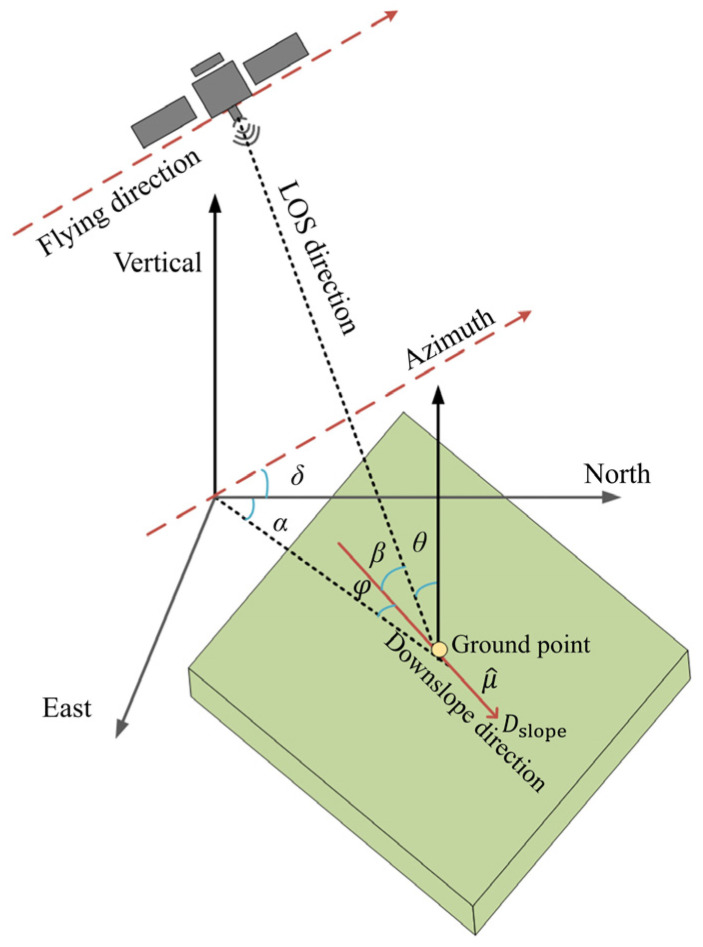
InSAR downslope displacement transformation model of landslide and SAR imaging geometry.

The relationship between the LOS displacement and the downslope displacement of the landslide was derived from the geometric relationships illustrated in Figure 5, taking into account the slope and aspect of the landslide.

(1) $$\hat{u}=\left[\begin{array}{c} -sin\alpha cos\varphi \\ -cos\alpha cos\varphi \\ sin\varphi \end{array}\right]$$

(2) $$cos\beta =\left(-sin\alpha cos\varphi \right)\left(-sin\vartheta cos\delta \right)+\left(-cos\alpha cos\varphi \right)\left(-sin\vartheta sin\delta \right)+sin\varphi cos\theta$$

(3) $$V_{slope}=\frac{V_{los}}{cos\beta }$$

where $D_{slope}$ is the displacement of the matter element on the landslide surface in the downslope direction [42], α denotes the slope aspect, δ is the angle between the azimuthal and due-north directions, φ represents the slope gradient, β is the angle between the slope and the LOS direction, and ϑ is the angle of incidence, which is calculated using the formula provided above.

## 4. Results and Analysis

### 4.1. Satellite InSAR Measurement Results

**Figure 6:**
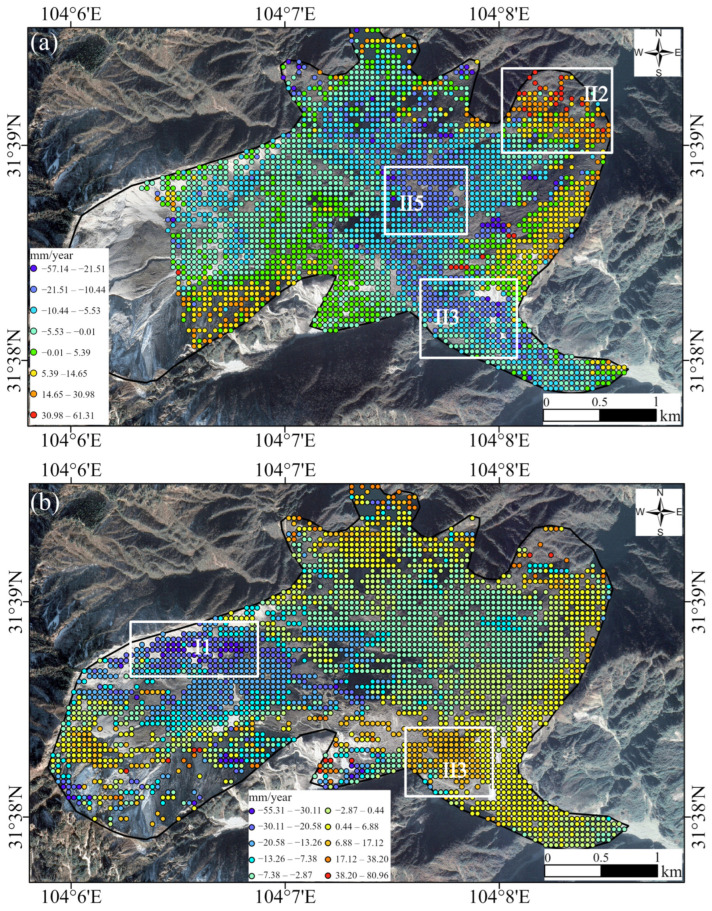
Deformation rate along the LOS direction in the radar coordinate system. (**a**) LOS direction deformation of path 128 (ascending). (**b**) LOS direction deformation of path 62 (descending). The areas within the white rectangular frames highlight regions with significant deformation changes. II1 represents the Northern Scarp Zone, composed of dolomite and slate, with substantial rockfall deposits. II2 refers to a barrier lake formed by rock debris along the Chuanlin Gully. II3 is primarily composed of black limestone fragments, which flowed downward along the Huangdongzi Gully, forming an accumulation zone. II5 serves as the Main Accumulation Zone, functioning as the primary deposition site for landslide materials.

As shown in Figure 6a,b, the LOS deformation rate results for the ascending and descending orbits exhibit distinct spatial characteristics. This difference is likely due to two factors. First, the varying looking directions of the ascending and descending SAR satellites result in different LOS deformation patterns. Second, the differences in observations may reflect the complex motion mechanisms of the DGBL. Notably, the presence of several moving blocks within the DGBL suggests that the heterogeneous spatial deformation implies varying motion directions among these blocks. Specifically, the trailing edge fault wall region of the DGBL, which was damaged by strong vibrations and pulling forces, appears to align more closely with the descending LOS direction rather than the ascending LOS direction. As a result, deformation in this region is detected only in the descending orbit results and not in the ascending orbit results.

From Figure 6a, it is evident that LOS-direction deformation from the ascending orbit is primarily concentrated in the central and front edge areas of the DGBL. Most regions in these areas exhibit movement away from the satellite, with the maximum annual deformation rate reaching 61.3 mm/year. In contrast, Figure 6b shows that deformation results from the descending orbit are more extensively distributed across the central and rear parts of the landslide. The majority of regions in these areas demonstrate movement toward the satellite, with the highest average annual displacement rate reaching approximately 80.9 mm/year.

Specifically, Figure 6a highlights the influence of the Chuanlin Gully in the northeastern corner of the landslide (marked by the white rectangular frame in region II2) on the surface kinematics of the DGBL. This area moves away from the satellite due to debris accumulation and subsidence, resulting in positive values in the ascending orbit. The main debris accumulation area (highlighted by the white rectangular frame in region II5) is located at the center of the landslide, while the primary debris accumulation outlet (shear outlet) is situated in the southeastern corner (white rectangular frame in region II3), which is also the lowest part of the entire landslide. Consequently, these two areas exhibit movement toward the satellite, accompanied by uplift from debris buildup, reflected as negative values in the ascending orbit.

Figure 6b shows that the LOS displacement results from the descending orbit display positive values in the southeastern corner of the landslide (see the white rectangle in region II3), indicating an uplift in this area. In the northern scarp area of the northwest corner of the landslide (white rectangle in region I1), the ground surface is clearly observed moving away from the satellite. This reflects the ongoing subsidence of fragmented rock masses caused by the landslide following the earthquake. These observations underscore the complex deformation patterns of the landslide, which are influenced by topographical features and debris dynamics, as captured by both ascending and descending Sentinel-1 InSAR observations.

### 4.2. GB-SAR Measurement Results

**Figure 7:**
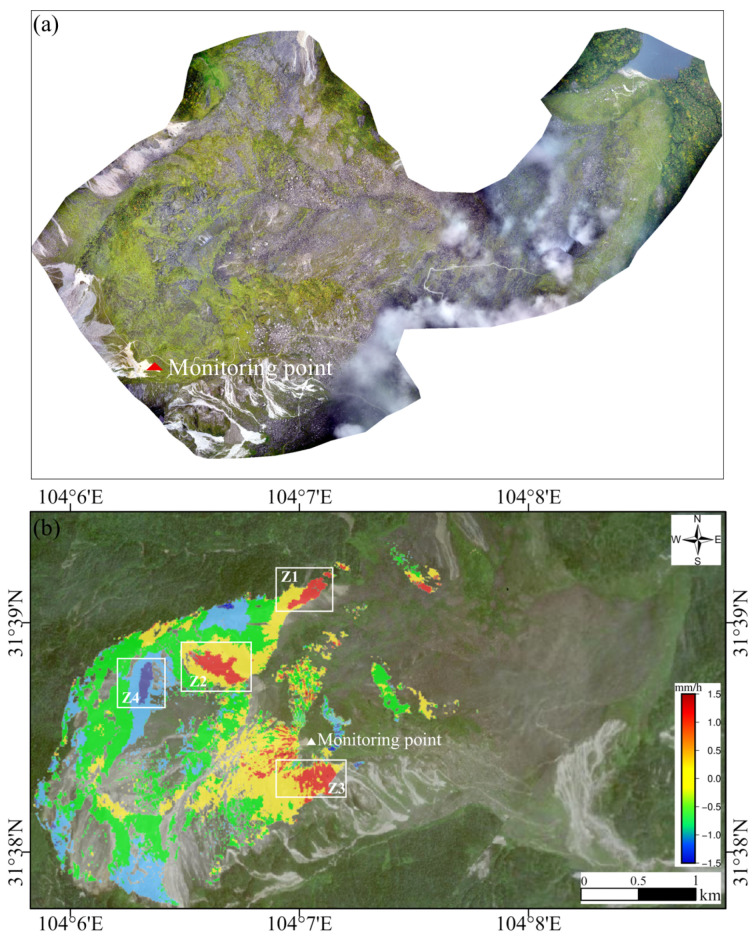
(**a**) The DOM of the DGBL. (**b**) The LOS displacement rate map of the DGBL obtained from the GB-SAR data (negative values indicate that the ground surface is moving away from the sensor). The areas Z1, Z2, and Z4 are located in the northern fault scarp area. Z3 is a slope rock detritus area, with the lithology mainly consisting of gray-black limestone, which flows downward along the Menkanshi Gully to form an accumulation area. The triangle marks the location of the GB-SAR monitoring site.

Figure 7a displays the DOM of the DGBL, highlighting the rugged terrain of the area. In the central accumulation and front edge regions, the presence of vegetation cover results in poor coherence. This has limited the effectiveness of ground-based InSAR technology in imaging the area, making it challenging to obtain reliable data encoding [43]. The deformation rate shown in the figure represents only a portion of the landslide area. The deformation detected by ground-based radar is primarily concentrated at the rear edge of the landslide, complementing the results of InSAR monitoring.

Figure 7b illustrates the displacement rate observed by the GB-SAR during the observation period. From Figure 7b, it can be seen that during the monitoring period, the LOS deformation results from the ground-based InSAR show positive values (i.e., moving away from the radar LOS) in areas near the radar center point and negative values (i.e., moving towards the radar LOS) in areas farther from the radar center point. This aligns with the trend of the landslide body moving towards the radar station and sliding downslope, consistent with its movement pattern.

Based on the overall monitoring results of the GB-SAR, the maximum deformation rate of the landslide reaches 1.5 mm/h. As shown in Figure 7b, the uplift rates in areas Z1, Z2, and Z3 were relatively high, with the maximum average deformation rate reaching 1.0 mm/h. Conversely, the subsidence rate in area Z4 was relatively high, with the maximum average deformation rate reaching −1.5 mm/h.

**Figure 8:**
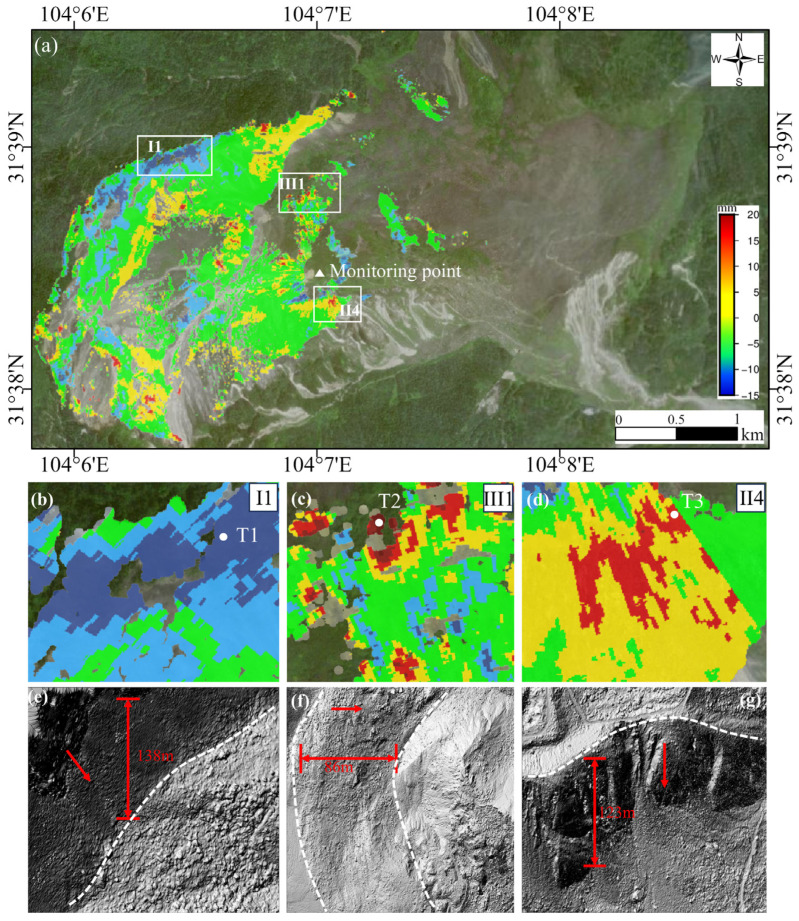
(**a**) Cumulative LOS displacement map of DGBL obtained from GB-SAR data. (**b**–**d**) Correspond to the deformation maps of regions I1, III1, and II4. I1 represents the Northern Scarp Zone, III1 represents the Secondary Slide Mass, and II4 represents the Menkanshi Gully Accumulation Zone. (**e**–**g**) Hillshaded Lidar DSM; red arrows indicate the maximum topographical gradient, and the white dashed line marks the gully boundary.

To better understand the deformation mechanism of landslides, the InSAR deformation results were analyzed with the assistance of lidar-derived fine topography. Figure 8e–g illustrates the hillshaded Lidar-derived DSM. The terrain in the I1 region, located at the rear edge where T1 is situated, is characterized by steep slopes with a height difference of 138 m and an average slope of 40°, oriented southeast. As shown in Figure 8e, the steep cliffs in this area, along with geological structural activities, appear to be one of the primary controlling factors for the deformation of rock detritus. The presence of these cliffs compromises the overall stability of the debris, leading to settlement and sliding under the influence of gravity.

In the III2 region, which represents the secondary debris accumulation area, the slope faces eastward, and the boundary line is distinctly visible in Figure 8f. This region has a height difference of 86 m and an average slope of 35°. Here, rock debris moves downstream due to gravitational forces, resulting in an overall uplift in the III1 region, where T2 is located. This phenomenon suggests that the deformation of rock detritus is influenced not only by slope and aspect but also by the accumulation of rock debris and gravitational forces.

In the II4 region, the main debris accumulation area where T3 is located, the height difference is 123 m, and the average slope is 45°. In this region, rock detritus slides along the sliding surface. Due to the convergence effect along the sliding direction on the southern side of the source area, part of the material from the main sliding body is pushed out of the groove and either ejected or spread out, forming a significant uplift in the area. This indicates that the deformation characteristics in the debris accumulation area are closely linked to the steepness of the terrain, the volume of rock debris accumulation, and local geological conditions. Overall, the deformation characteristics of rock detritus are primarily controlled by the combined effects of slope, aspect, geological structure, and rock debris accumulation. Notably, local geological conditions in steep areas play a decisive role in determining the stability and deformation patterns of rock detritus.

To quantitatively assess the long-term temporal evolution captured by ground-based radar, we conducted a time series analysis of three representative feature points, T1, T2, and T3, located within the unstable region of the landslide mass. Feature point T1 is situated in the I1 region, where the incidence angle of the GB-InSAR radar waves is favorable, and there is a distinct linear boundary between the exposed rock surface and the vegetated surface of the residual rock mass. Feature point T2 is located in the III1 accumulation area, positioned in the middle of the unstable zone of the landslide mass, which is characterized by high fragmentation and steep topography. Feature point T3 is located in the II4 accumulation area of the Menkanshi Gully, where rock detritus flows down the gully and forms an accumulation zone.

**Figure 9:**
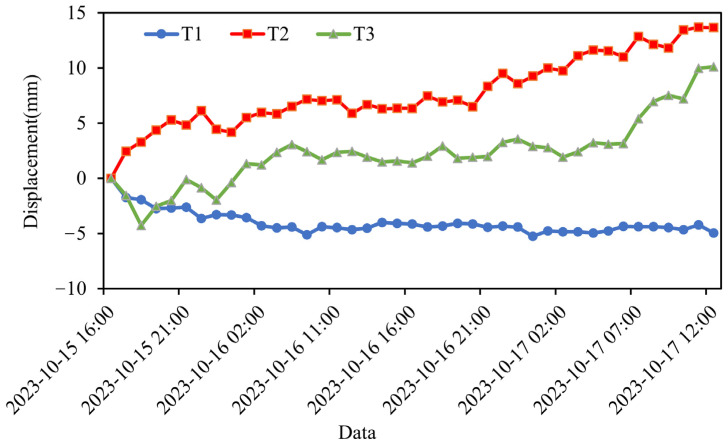
Time series of LOS displacement of three feature points (T1–T3).

The selection of these three feature points, each with distinct topographic characteris-tics, helps to better understand the specific movement patterns and potential influencing factors in different regions of the DGBL. This analysis aims to provide critical insights for post-earthquake recovery efforts and the development of early warning systems. Figure 8b–d illustrates the positions of these feature points, while Figure 9 presents the time series deformation graphs for these points within the landslide.

The data reveals that T2 exhibits the largest cumulative deformation, reaching 13.7 mm, followed by T3 with a cumulative deformation of 10.1 mm. In contrast, T1 shows the smallest deformation at −4.9 mm. T1 is located in the steep scarp area on the northern side of the landslide and is farther from the radar system during the observation period. Although the movement rate in the region where T1 is located is relatively high, the overall deformation time series indicates that T1 remains essentially stable. On the other hand, feature points T2 and T3 moved toward the radar system during the observation period, displaying similar movement trends. Notably, T2, located in the main landslide accumulation area, demonstrates significant cumulative deformation, with deformation continuing to increase over time.

### 4.3. Downslope Displacements of DGBL

**Figure 10:**
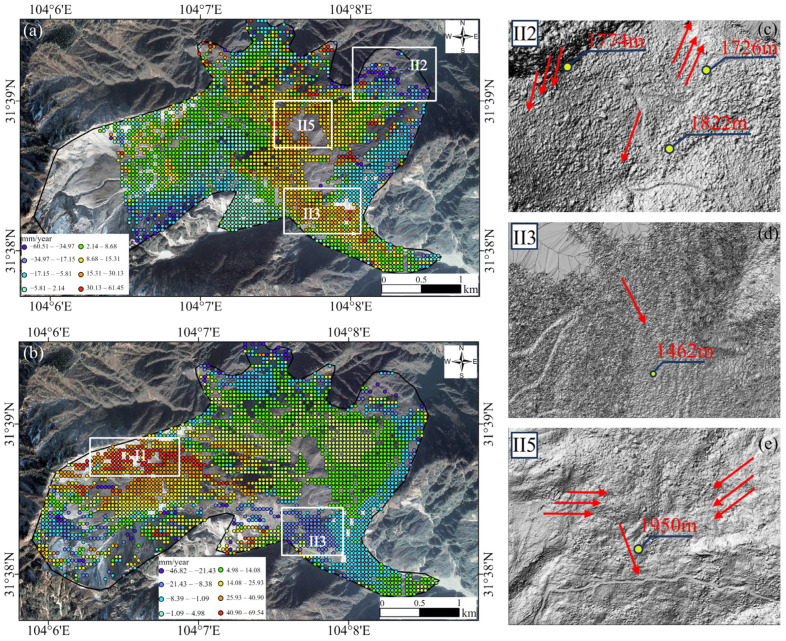
(**a**) Ascending orbit annual downslope deformation rate. (**b**) Descending orbit annual downslope deformation rate (areas within rectangular frames indicate regions with significant deformation changes). (**c**–**e**) Hillshaded LiDAR DSM, with red arrows indicating the maximum topographical gradient. I1 represents the Northern Scarp Zone, while II2 corresponds to the Chuanlin Gully Barrier Lake Zone. II3 denotes the Huangdongzi Gully Accumulation Zone, and II5 represents the Main Accumulation Zone.

Based on the geometric characteristics observed in the SAR images, the ascending and descending LOS deformation results from Sentinel-1 were projected onto the downslope direction of the landslide, with the downslope direction defined as positive. Figure 10a illustrates the deformation velocity field along the downslope direction for the ascending orbit, while Figure 10b shows the same for the descending orbit. The deformation rates along the downslope direction, derived from the ascending and descending datasets, range from −60.5 mm/year to 61.4 mm/year and −46.8 mm/year to 69.5 mm/year, respectively. An analysis revealed that the middle and lower parts of the landslide exhibited higher deformation rates in both datasets. Four significant deformation areas (I1, II2, II3, and II4) were identified based on deformation velocity magnitude, as highlighted in Figure 10a,b. The slope direction deformation rates were consistent with the LOS deformation rates, confirming the reliability of the identified deformation zones.

In the ascending orbit results, region II2, located at the front edge of the DGBL, is influenced by steep slopes on the northeastern side of Pingliangzi Mountain and Chuanlin Gully, resulting in multi-directional movements. Sediment flows along Chuanlin Gully form barrier lakes, with debris moving downslope to the southwest, reaching an elevation of 1822 m. Region II5, significantly affected by topography, experiences the weathering of rock debris surfaces. During continuous rainfall, water infiltrates cracks, acting on deeper accumulations and causing material to slide downslope. The blocking effect of Pingliangzi Mountain concentrates material in this region, increasing its elevation to 1950 m and resulting in significant deformation velocity.

In the descending orbit results, region I1, located at the rear edge of the DGBL, also exhibited significant deformation. Geological characteristics, combined with gravity and precipitation, contributed to the high downslope deformation rate in this area. This highlights the diverse modes of downslope deformation in large-scale landslide accumulations like the DGBL, emphasizing the importance of slope deformation in understanding landslide dynamics.

Region II3, identified in both the ascending and descending results, also shows significant deformation. With an elevation of 1462 m and a steep slope angle of 50°, the terrain in this area is highly susceptible to deformation. Loose accumulations are easily affected by rainfall and scouring from Huangdongzi Gully, leading to significant deformation and the formation of a small accumulation body at the scarp outlet. Precipitation accelerates weathering, with water infiltrating cracks in the debris and acting on deeper layers, further driving deformation under the influence of gravity and rainfall.

This analysis indicates that the deformation evolution of the DGBL is influenced by multiple factors, including topography, earthquakes, and rainfall. Intense seismic activity, such as the Wenchuan earthquake, caused ground cracks, reduced basal friction, and weakened landslide strength. The earthquake also generated large amounts of loose debris, which, combined with rainwater infiltration, increased pore water pressure and reduced the shear strength of the sliding mass. These conditions, along with steep topography and exposed rock debris, have contributed to the continuous deformation of the DGBL.

## 5. Discussion

Our InSAR observations indicate that the LOS deformation rates closely align with the downslope deformation results, with significant deformation at the Huangdongzi Gully (scarp outlet) showing consistent patterns. Additionally, GB-SAR monitoring has provided detailed insights into the rear edge of the rock detritus and the middle accumulation area, yielding higher-resolution deformation rate results. The next steps will focus on two aspects: (1) a comparison between the observation results of satellite-based and ground-based InSAR, and (2) a comparison with the findings from the existing literature.

### 5.1. Comparison of Satellite- and Ground-Based InSAR Measurements

**Figure 11:**
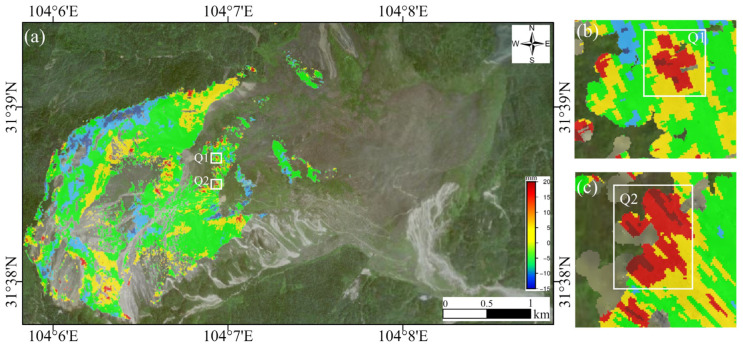
(**a**) The Q1 and Q2 regions, which are located in areas of significant deformation. (**b**,**c**) Correspond to the deformation rate maps of Q1–Q2, respectively.

The comparison of the satellite-based and ground-based InSAR monitoring results in the research region is conducted from the perspective of LOS deformation rates, focusing on the common areas Q1 and Q2, as shown in Figure 11. The GB-SAR monitoring period spans from 15 to 17 October 2023, while the satellite-based InSAR monitoring period is from June to December 2023. To ensure consistency, all the deformation rates are converted to mm/day.

**Figure 12:**
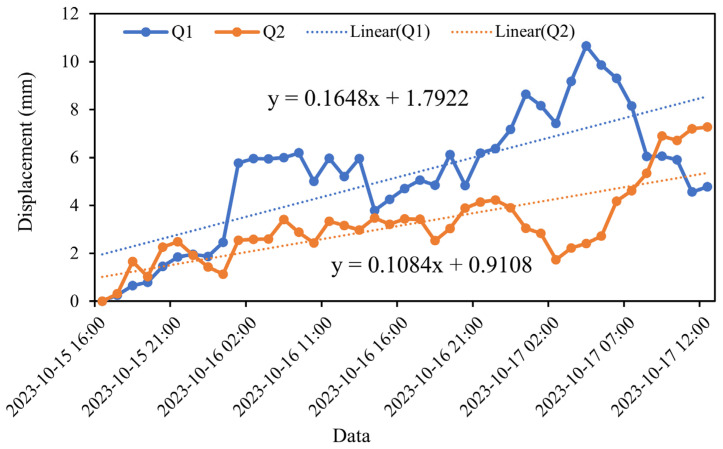
GB-SAR rate plots for the Q1 and Q2 regions. The solid line in the figure represents the cumulative displacements of Q1 and Q2.

The GB-SAR LOS deformation rate results, presented in Figure 12, show rates of 4.0 mm/day for Q1 and 2.5 mm/day for Q2. In contrast, the LOS deformation rates obtained using the SBAS-InSAR technique for the ascending orbit are −0.4 mm/day for Q1 and −0.5 mm/day for Q2. For the descending orbit, the LOS deformation rates are −0.3 mm/day for Q1 and −0.2 mm/day for Q2.

This comparison highlights a significant difference between the deformation rates observed by GB-SAR and satellite-based InSAR. The GB-SAR results indicate much higher deformation rates in both Q1 and Q2, while the satellite-based InSAR results show relatively minor deformation rates in the same regions. This discrepancy may be attributed to the higher spatial and temporal resolution of GB-SAR, which allows for more detailed monitoring of localized deformation, particularly in areas with rapid or small-scale movements. Conversely, satellite-based InSAR provides a broader, long-term perspective but may underestimate deformation rates in regions with rapid changes or complex terrain.

Second, the observed deformation trends reveal that the deformation patterns in certain areas, as detected by satellite-based and ground-based InSAR, are consistent. Within the selected unstable region, the deformation trend in Area I1 (Figure 8) aligns with the results from descending orbit monitoring, with both indicating movement away from the radar system.

Three primary factors contribute to the discrepancies in the deformation rates derived from the two approaches: First, there is an inherent limitation in the data due to the insufficient integration of the two methodologies at the data level, largely stemming from the minimal overlap between the monitoring periods of GB-SAR and satellite-based SAR. Second, differences in spatial resolution play a role. The Sentinel image data used in satellite-based InSAR monitoring has a spatial resolution of 5 m × 20 m, whereas GB-SAR data provides a much finer spatial resolution of 0.3 m × 1°. Consequently, the monitoring results from satellite-based InSAR are theoretically less detailed than those from GB-SAR. Lastly, satellite InSAR technology has its own inherent limitations. It is vulnerable to atmospheric interference, and factors such as Sentinel-1 data quality, DEM accuracy, and other variables can influence the results. Additionally, InSAR is affected by perspective distortion, overlay masking, shadowing, and other effects, particularly in areas with significant terrain relief.

Both large-scale satellite monitoring and small-scale ground-based monitoring offer unique value and applicability in analyzing surface deformation and monitoring potential geological hazards. It is crucial to apply these techniques to other large-scale landslides. Despite the noted differences, the two methods demonstrate consistency in deformation trends within certain sub-regions.

### 5.2. Comparison with Existing DGBL InSAR Monitoring Results

Utilizing the various techniques outlined in Section 2, several research groups (Chen et al., 2014; Dai et al., 2016; Luo et al., 2020; Yu et al., 2021) [6,7,8,9] have obtained specific monitoring results for the DGBL. The sliding rate of the landslide reported in this study is noticeably slower during the radar image observation period compared to the rate documented in the literature from 2008 to 2010 [6]. Moreover, the spatial distribution of the sliding rate and deformation area closely aligns with the observations reported in the literature from 2015 to 2020 [7,8,9]. This suggests that the landslide has been under continuous monitoring since the Wenchuan Earthquake. The findings indicate that the deformation rate of this landslide decreased sharply following the Wenchuan Earthquake and has varied over time, yet it remains generally stable.

Furthermore, our method enables the monitoring of a broader range of deformations. Both descending orbit data and GB-SAR data have detected the landslide scarp area (Zone I), which was previously undetected by InSAR technology. Notably, the northern scarp area, I1, exhibits significant deformation. Luo and his team obtained large-scale landslide deformation results using descending orbit Sentinel-1A data, while we utilized data from both Sentinel-1A and Sentinel-1B. Dai and his team identified a main steep slope in the southeastern segment of the co-seismic large-scale landslide, with a secondary steep slope behind it that clearly defines the sliding area with maximum LOS displacement. The thickness of the accumulated debris can reach up to 500 m, and the sliding mass exhibits an easterly movement along the Huangdongzi gully. Our findings also reveal accumulation along the Huangdongzi gully, with an accumulation rate exceeding 80.0 mm/year. This consistency has been confirmed by Luo and his team, and Yu and his team, further validating the instability of the slope in this area.

The most significant advantage of GB-SAR over the other four datasets is its sub-millimeter monitoring accuracy. According to the cumulative displacement map, the two-day cumulative deformation in the monitoring area ranges from −15.7 mm to 20.0 mm, with the P3 feature point in the main landslide accumulation area exhibiting the highest cumulative displacement of up to 20.0 mm. However, due to insufficient coherence caused by factors such as the incidence angle and scattering coefficients, no GB-InSAR data are available for the middle accumulation zone and the front edge of the landslide region.

Given that satellite-based and ground-based InSAR operate on different spatial and temporal scales, the integration of these technologies leveraging their respective characteristics and differences enables the acquisition of critical ground displacement measurements with high spatial and temporal resolution.

## 6. Conclusions

This study integrated satellite- and ground-based InSAR observations to develop a high-precision surface motion monitoring system for large-scale landslides. By utilizing both ascending and descending orbit data, we accurately estimated the downslope deformation rates of DGBL, providing strong evidence for landslide deformation trends. The spatiotemporal analysis of surface deformation in the DGBL region revealed that the area remains in a state of continuous movement. The results indicate significant downslope deformation in the DGBL region, with maximum LOS deformation rates of 80.9 mm/year and 61.3 mm/year for descending and ascending orbits, respectively, while ground-based radar monitoring recorded a maximum deformation rate of 1.5 mm/h. A comprehensive analysis identified steep and rugged terrain, along with fragile and densely jointed lithology, as the primary driving factors for the sustained deformation of the landslide. In the future, the integration of additional SAR data sources may enable the acquisition of three-dimensional landslide displacement data, facilitating the long-term observations of the landslide’s evolution as it moves toward recovery. Moreover, the utilization of GB-SAR for long-term time series data monitoring in the study area allows for the exploration of deformation monitoring under conditions of non-continuous observations and a deeper investigation into the complementary relationship with satellite InSAR.

Our study provides a reference for monitoring and analyzing the evolution of similar post-seismic landslides. High-precision deformation monitoring data enables the timely identification of potential landslide risk areas, offering a scientific foundation for the development of disaster prevention and mitigation strategies. Furthermore, the integration of satellite- and ground-based InSAR technologies delivers robust technical support for early warning systems targeting geological hazards, with practical applications in landslide risk assessment, disaster warning, and emergency response.

## Figures and Tables

**Table 1:** Basic parameters of SAR data.

Satellite	Orbit Direction	Incident Angle (°)	Heading Angle (°)	Number of Images	Acquisition Dates
Sentinel-1A	Ascending (P128)	44.2	−12.8	80	20210110–20231226
Sentinel-1A/B	Descending (P62)	37.6	192.8	111	20210105–20231221

**Table 2:** Basic parameters of GB-SAR system.

Monitoring Accuracy (mm)	Maximum Viewing Angle (°)	Acquisition Interval (min)	Acting Distance (km)	Pixel Size	Total Observation Time (h)
0.1	360° × 60°	6	5	0.3 m × 1°	45

## Data Availability

The data presented in this study are available upon request from the corresponding author.
